# Pharmacokinetics of rectal levetiracetam as add-on treatment in dogs affected by cluster seizures or status epilepticus

**DOI:** 10.1186/s12917-018-1522-0

**Published:** 2018-06-18

**Authors:** Giulia Cagnotti, Rosangela Odore, Giulia Gardini, Stefano Amedeo, Iride Bertone, Giulia Guerriero, Laura Lentini, Elena Dappiano, Antonio D’Angelo

**Affiliations:** Department of Veterinary Science, University of Turin, Via Largo Braccini 2, 10095 Grugliasco, Turin, Italy

**Keywords:** Epilepsy, Pharmacokinetics, Neurology, Emergency

## Abstract

**Background:**

Levetiracetam can be used for seizure control alone or in combination with other antiepileptic medications. A previous study achieved the minimum targeted serum drug concentration after rectal administration of levetiracetam in healthy dogs. The purpose of the present study was to determine the pharmacokinetics of rectal LEV in dogs presented for cluster seizures or status epilepticus and potentially in treatment with other anti-epileptic drugs. Furthermore, preliminary information on response to this treatment as add-on to the standard treatment protocol is reported.

**Results:**

Eight client-owned dogs were enrolled. Plasma levetiracetam concentrations (measured at 0, 30, 60, 90, 120, 180, 240, 360, 720, and 1440 min after drug administration) reached the minimum target concentration (5 μg/ml) at 30 min in all but one patient. At T1 (30 min) the mean concentration was 28.2 ± 15.5 μg/ml. Plasma concentrations remained above the targeted minimum concentration in all patients until 240 min and in 7/8 until 360 min. Six out of eight patients experienced no seizures in the 24-h period after hospitalization and were classified as “responders”.

**Conclusions:**

Minimum plasma levetiracetam concentration can be reached after rectal administration of 40 mg/kg in dogs affected by cluster seizures and status epilepticus and concurrently receiving other antiepileptic drugs. These preliminary results may encourage the evaluation of rectal levetiracetam as an additional treatment option for cluster seizures and status epilepticus in a larger number of dogs.

**Electronic supplementary material:**

The online version of this article (10.1186/s12917-018-1522-0) contains supplementary material, which is available to authorized users.

## Background

Canine epilepsy is among the most common neurological diseases in dogs [1]. Cluster seizures (CS) are defined as the occurrence of two or more seizures within a 24-h period, with complete recovery of the state of consciousness in between; status epilepticus (SE) refers to seizure activity lasting for 5 min or longer or when there’s no complete recovery of the state of consciousness between two seizure events [2]. CS and SE are potentially life-threatening neurological emergencies and are considered risk factors for spontaneous death or euthanasia of dogs affected by epilepsy [3–7]. As such, these conditions are a frequent reason for presentation to emergency veterinary services [8, 9]. To date, first line therapy is intravenous or rectal administration of diazepam during the seizure event [10–12]. Unfortunately, not all dogs will respond to benzodiazepines and can experience refractory SE. Moreover, prolonged seizure activity is known to decrease the effectiveness of benzodiazepines in human medicine [13]. Levetiracetam (LEV), a pyrrolidone derivative, is a novel antiepileptic drug that was approved in the United States in 1999 for the oral treatment of partial onset seizures in humans [14]. Its mechanism of action is not fully understood, but it seems to differ completely from other antiepileptic medications (AEDs). LEV is thought to act by binding the synaptic vesicle protein 2A on the presynaptic terminal, thus modulating exocytosis of neurotransmitters [15]. Due to its favorable therapeutic profile, LEV has been increasingly used for seizure control either alone or in combination with other first line AEDs in veterinary medicine [16]. In their study published in 2014, Peters and colleagues found a rapid rise in serum LEV concentrations associated with maintenance of values above the targeted minimum concentration up to 9 h after rectal administration of a LEV formulation in healthy dogs [17].

Based on these premises, the aim of this pilot study was to determine the pharmacokinetics of LEV administered per rectum in dogs presented for CS or SE and possibly already in treatment with other long-term AEDs. We hypothesized that LEV administered per rectum would achieve the targeted minimum plasma drug concentration in patients affected by CS and SE. Furthermore, we report the response to treatment as preliminary information on the potential association of LEV administered per rectum as an adjunct to standard treatment in patients referred for CS and SE.

## Methods

### Animals

The study was approved by the Bioethics Committee of the University of Turin (protocol #9834 dated 25/02/2016). The owners gave their written, informed consent to their dog’s enrollment in the study. Client-owned dogs (minimum weight 20 kg) presented with CS or SE to the Veterinary Teaching Hospital (VTH), Department of Veterinary Science of Turin, between October 2016 and April 2017 were eligible for inclusion. SE was defined as a seizure event lasting more than 5 min or two or more seizures without complete recovery of consciousness in between. CS were defined as two or more seizures occurring within a 24-h period. Dogs were excluded if they were already in treatment with LEV for long-term seizure control or if further diagnostic tests indicated reactive seizures.

### Study design

At the time of presentation to the VTH, seizure activity was immediately controlled by standard care comprising rectal administration of diazepam (at a dosage of 1–2 mg/kg if the dog was seizuring at presentation) followed by IV administration of phenobarbital (4–5 mg/kg q8h). As soon as possible after hospitalization, and always within 2 h from the presentation, LEV suspension (at a dosage of 40 mg/kg) was administered per rectum. The dosage was based on the results of a previous study [17]. A rigid, sterile, male dog urinary catheter (BUSTER Disposable Dog Catheter, Buster, Kruuse, Germany) was cut to 5 cm length and inserted approximately 3 to 4 cm into the rectum. A syringe was then connected to inject the drug. The catheter was flushed with air immediately after the injection to ensure the administration of the remaining portion of LEV in the catheter. After removal of the catheter from the rectum, the anus was held closed for 5 min to prevent drug expulsion. The procedure was performed by the same investigator (G.C.) in all patients.

Venous blood samples were obtained immediately before drug administration (T0), and at 30 (T1), 60 (T2), 90 (T3), 120 (T4), 180 (T5), 240 (T6), 360 (T7), 720 (T8), and 1440 (T9) min thereafter. Blood samples were collected in ethylenediaminetetraacetic acid tubes, and plasma was separated immediately after sampling by centrifugation at room temperature (3500 × *g*, 5 min) and then frozen at − 20 °C until analysis. Patients were assessed for signs of adverse reactions specifically attributable to LEV administration (decreased appetite and vomiting) by the same investigator (G.C.) at each time point and between the experimental time points by the intensive care unit veterinarians.

For the assessment of treatment efficacy, dogs were defined as “responders” if no further epileptic seizures occurred during the 24-h observation period between hospital admission and discharge; “non-responders” were dogs that experienced an additional epileptic seizure despite LEV administration in addition to the above-mentioned protocol in the 24-h period.

### Levetiracetam suspension

Pure LEV powder (Levetiracetam European Pharmacopoeia Reference Standard, Sigma-Aldrich, Saint Louis, MO, USA) was purchased and mixed with sterile water to make a suspension with a LEV concentration of 200 mg/ml. This was done to reduce the volume of solution for rectal administration and minimize the risk of accidental evacuation of the drug. The suspension was formulated and replaced every month. LEV suspension was stored at room temperature away from direct light and always vigorously shaken to suspend the powder before administration.

### Determination of plasma levetiracetam concentrations

Levetiracetam powder and all other reagents were purchased from Sigma-Aldrich. LEV was analyzed on a high-performance liquid chromatography (HPLC) system (Dionex Thermo Fischer Scientific, Sunnyvale, CA, USA) and separation was performed on a C18, 5 μm, chromatography column (Dionex Thermo Fischer Scientific) protected by a security guard precolumn. Chromatographic run was carried out at 35 °C for 20 min with a step gradient starting at 0 min with 95% solvent A (H_3_PO_4_ 0.423% in water) and reaching 100% solvent B (acetonitrile) at 12 min. Detection was performed at λ = 210 nm. The limit of detection was 1 μg /ml. For LEV extraction, 500 μl of plasma were mixed with 10 μl of HClO_4_ and 500 μl of methanol. The samples were then vortexed for 2 min and centrifuged at 17,000 × *g* for 5 min. Forty microliters of supernatant were then analyzed by HPLC. The unknown concentrations of LEV in samples were quantified by comparing the signal to standard calibration curve (R^2^ = 0.9947). The recovery percentage was 99.2 ± 4.9%.

### Data analysis

Continuous variables, including patient age and weight at inclusion, were reported as median (minimum – maximum) [min – max]. Pharmacokinetic parameters were estimated by plotting LEV concentrations versus time. Data were analyzed using a Chromelion 6 Chromatography data system (Chromelion 6 Chromatography data system, Thermo Fischer Scientific), and statistical analysis was performed using GraphPad InStat 3.0 (GraphPad InStat 3.0, GraphPad Software, La Jolla CA, USA). Parameters were area under the curve (AUC), maximum concentration (C_max_), time to maximum concentration (T_max_), and half-life (t ½). Non-compartmental analysis was performed with AUC calculated using the linear trapezoidal method. The Shapiro-Wilk test showed normal distribution of the dataset; data were reported as mean ± standard deviation (SD).

## Results

A total of 36 dogs were presented for CS or SE to the VTH between September 2016 and April 2017. Eight dogs met the inclusion criteria and were included in the study. The other 28 patients were excluded because: body weight less than 20 kg (16/28), no consent given by the owners for inclusion in the study (7/28), long-term oral LEV administration for seizure control (3/28), and diagnosis of reactive seizures (2/28).

Among the eight dogs included in the study, five were intact females and three were males (two intact and one neutered); the median age and weight at presentation were 75 months (range, 43–126) and 34 kg (range, 24–52), respectively. Detailed information on signalment and history are reported in an additional file (see Additional file 1). Blood work comprising complete hematology and biochemistry panel, bile acid stimulation test, and blood ammonia concentration resulted within normal limits. Four dogs were diagnosed with suspected idiopathic epilepsy based on signalment, history, and normal interictal neurological examination. Magnetic resonance imaging (MRI) and cerebrospinal fluid (CSF) analysis were available for only one patient and were unremarkable. Signalment, history, and abnormal interictal neurological examination aroused suspicion of structural epilepsy in the four other patients. In two of these cases a neoplastic lesion (suspected glioma) was confirmed by MRI investigation. A space-occupying lesion was suspected in the other two patients based on signalment and the findings of neurological examination. The neurological examination was performed by a board-certified neurologist (A.D.A.) or a neurology resident (G.C.) under supervision of the board-certified neurologist.

At the time of inclusion in the study, four out of eight dogs had been receiving phenobarbital (PB) therapy for long-term seizure control; two were concurrently receiving potassium bromide (KBr) and one patient was on treatment with Imepitoin. The remaining three dogs had not received any previous AED therapy (see Additional file 1). The patients receiving PB alone or in combination with KBr had been in treatment for longer than the period needed to achieve steady state of the drugs (14 days and 1–3 months, respectively). PB dosage varied from 2.6 to 6.3 mg/kg q12h, (median, 3.6 mg/kg q12h); the KBr dosage was 40 and 27 mg/kg q24h in each of the two dogs, respectively. Imepitoin was administered at a dosage of 15.7 mg/kg q12h.

**Fig. 1:**
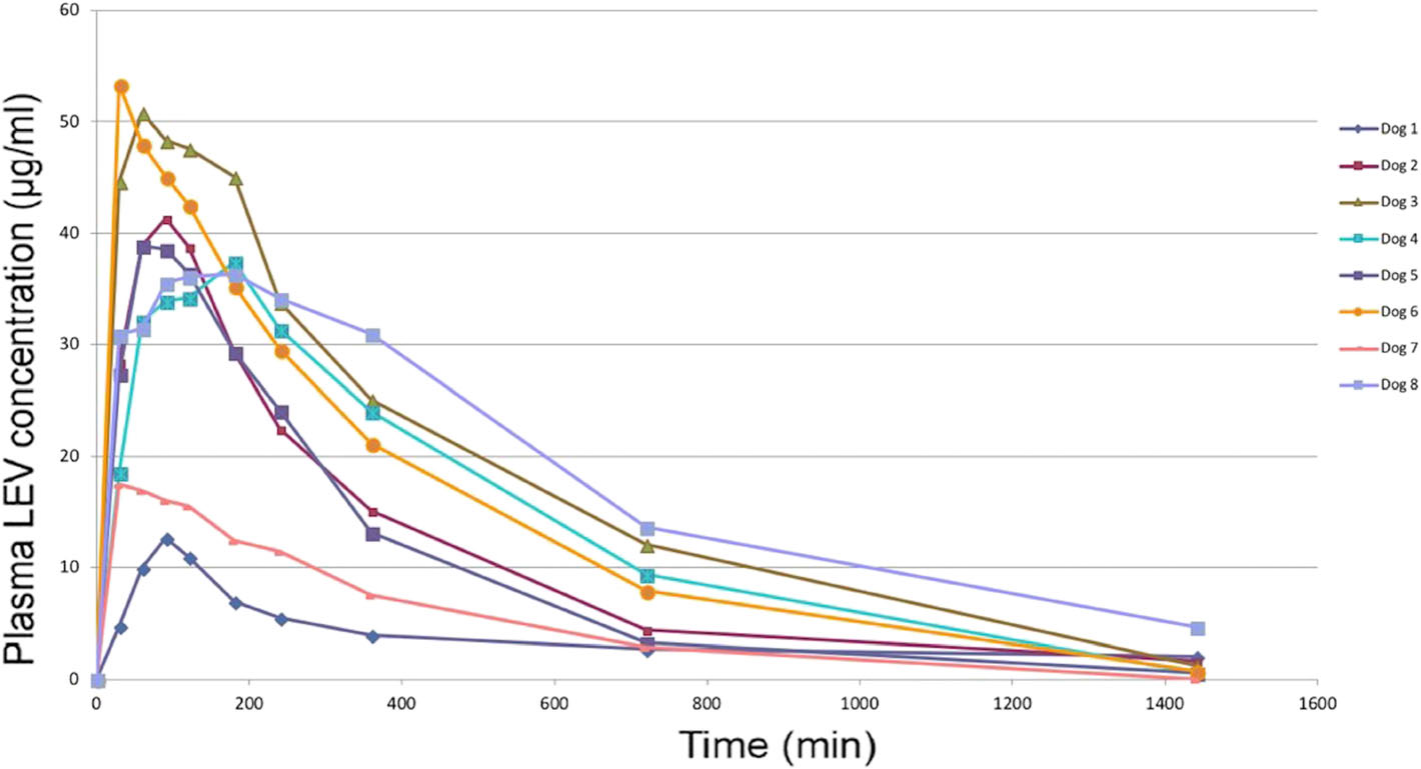
Time course of plasma LEV concentration after rectal administration

Plasma LEV concentrations at the nine time points are shown in Fig. 1. At the first experimental time point (T1) the mean concentration was 28.2 ± 15.5 μg/ml (*n* = 8). At this time point (T1), plasma LEV concentrations reached the minimum target concentration of 5 μg/ml in all but one patient, in which it was slightly lower than the target (4.7 μg/ml). Plasma LEV concentrations remained above the minimum target range in all patients until T6 and in 7/8 (88%) patients at T7.

The plot of plasma LEV concentration versus time showed a lower peak concentration and a more rapid decrease in LEV concentration over time in two patients. Pharmacokinetic analysis of the data from the eight dogs revealed a C_max_ of 36.0 ± 14.4 μg/ml, with a T_max_ of 90 ± 60 min. The t ½ was 251.7 ± 75.6 min and the AUC 227.8 ± 131.8 μg-h/ml.

Six out of eight patients (75%) experienced no further seizures during the 24-h observation period and between hospital admission and discharge. Two patients (25%), diagnosed with confirmed and suspected idiopathic epilepsy, respectively, and both with lower peak concentrations and a more rapid decrease in LEV concentration over time, were classified as “non-responders”. They required further medications (constant rate infusions of diazepam in one and constant rate infusion of propofol in the other) for seizure control.

Considering the different outcomes, a post-hoc analysis was carried out with the patients grouped into “responders” (*n* = 6) and “non-responders” (*n* = 2). The results are shown in Table 1.

**Table 1:** Pharmacokinetics parameters

	C_max_ (μg/ml)	T_max_ (min)	AUC_0-t_ (μg-h/ml)	t ½ (min)
“Non-responders” (*n* = 2)	12.7 and 17.53	90 and 30	89.63 and 105.24	153 and 249
“Responders” (*n* = 6)	43.02 ± 7.27	100.2 ± 64.8	337.94 ± 83.41	268.6 ± 75.8

No adverse effects specifically attributable to LEV administration were noted at any time point

## Discussion

To our knowledge, this is the first study to evaluate LEV concentration after rectal administration in dogs presenting with CS or SE and potentially receiving concurrent therapy with other AEDs for long-term seizure control. In line with the observations reported by Peters and colleagues [17], our results show that the targeted minimum plasma LEV concentration can be achieved with rectal administration of 40 mg/kg. In the majority of cases, plasma concentrations reached the minimum targeted concentration after rapid absorption, already at the first blood sample taken 30 min after administration of the drug. A therapeutic range of LEV specific for dogs has not yet been established. The values of 5–45 μg/ml typically employed in veterinary medicine are deduced from human medicine [18]. The therapeutic range is highly variable, however, even in human patients, and mainly in correlation with age [19].

Peters and colleagues highlighted the potential risk of lower LEV absorption after rectal administration if palpable fecal material is present in the rectum [17]. The lower values of C_max_ and T_max_ for the two dogs in our series may be associated with less absorption of the drug due to the presence of fecal material. Another possible explanation for the different results is the concurrent long-term administration of PB. Indeed, LEV undergoes predominant renal excretion as unchanged drug (47 and 58% in female and male dogs, respectively). The remaining percentage of the drug is metabolized as acid metabolites and hydroxylated metabolites by hydrolysis and oxidation, respectively. This latter route of degradation was found to be induced by PB in rats and dogs [20]. Further investigations on dogs confirmed that chronic PB administration alters the metabolism of LEV, resulting in lower concentrations and more rapid renal clearance of LEV when administered per os [21, 22]. In all studies performed in veterinary medicine, only 21 days of PB administration were proven sufficient to increase metabolism of LEV, so the chronic PB administration is an unlikely explanation for the results in these two dogs. Unfortunately, we did not check for the presence of fecal material since we wanted to replicate the conditions in which rectal administration is performed in clinical settings. While this could be the most plausible explanation, we are unable to determine whether the lower drug absorption was due to any fecal material potentially present at the time of administration. This issue represents a limitation of the present study. Further studies are needed to evaluate the pharmacokinetics of rectal LEV in patients concurrently receiving PB, while excluding the confounding factor of feces present in the rectum. If this assumption is confirmed, it could also be interesting to assess the feasibility and safety of higher doses of LEV administered per rectum in patients under PB therapy.

One of the two dogs classified as “non-responders” had been diagnosed with idiopathic epilepsy at the time of inclusion in the study. According to the revised definition of pharmacoresistant epilepsy issued by the International League Against Epilepsy in human medicine in 2010 [23], this patient can be classified as pharmacoresistant, and so this condition could explain the patient’s non-responsiveness to treatment. Since seizure frequency was not recorded by the owner of the second non-responder patient diagnosed with suspected idiopathic epilepsy, it is impossible to establish whether this dog can be classified as pharmacoresistant as well. If LEV efficacy can be demonstrated in a larger number of cases, achievement of the targeted minimum LEV plasma concentration with rectal administration in epileptic dogs might allow at-home use of this formulation for better seizure control. The usage of IV/oral LEV in so-called “pulse treatment” for cluster seizures is well known [16, 18]. Nevertheless, in dogs experiencing seizures, the administration of oral medications may be delayed by the post-ictal phase, potentially leading to further seizure events. The rectal route of administration would avoid this delay and improve seizure control.

In our study, we formulated LEV suspension at a concentration of 200 mg/ml to reduce as much as possible the volume of medication introduced into the rectum, thus preventing induction of defection and subsequent accidental expulsion of the drug. The LEV suspension was made using pure LEV powder for scientific reasons. We also made LEV suspension from commercially available LEV tablets and found no differences in the chemical purity of the two formulations (data available from the authors on request).

The main limitations of the present study are the small patient series, the concomitant use of other AEDs, and the absence of a control group of patients for comparison. The designation of “responder” and “non-responder” was in reference to the combination of medications administered, and therefore it is not possible to discern any potential effect of rectal LEV administration. Therefore, we cannot conclude that rectal LEV is effective in preventing the onset of further seizures in patients with CS or SE. Nonetheless, these preliminary pharmacokinetic data are promising and are consistent with those reported by Peters and colleagues. Given the postulated enhancement of the anticonvulsive effects of benzodiazepines and the lack of side effects, such as cardiac and respiratory depression typical of other AEDs, [24] LEV can offer a potentially useful add-on to the treatment of seizure activity in dogs once its efficacy has been confirmed in a greater number of cases. Further studies are needed to confirm or confute our preliminary hypothesis. A future area of focus of this project is to better evaluate the efficacy of rectal LEV in a larger number of cases.

## Conclusions

In conclusion, our findings show that targeted minimum plasma LEV concentration can be reached after rectal administration of 40 mg/kg in dogs with CS or SE. These preliminary results, if confirmed, may allow for the use of rectal LEV as an additional treatment option for CS and SE in dogs.

## Additional file

Additional file 1: Signalment and history information of patients included. Information on signalment, seizure frequency, diagnosis (if achieved) of patients included in the study. (XLSX 27 kb)

## Abbreviations

AEDs: Antiepileptic medications
AUC: Area under the curve
C_max_: Maximal concentration
CS: Cluster seizures
CSF: Cerebrospinal fluid
HPLC: High performance liquid chromatography
KBr: Potassium bromide
LEV: Levetiracetam
MRI: Magnetic resonance imaging
PB: Phenobarbital
SE: Status epilepticus
T_max_: Time point at which C_max_ is measured
VTH: Veterinary teaching hospital
